# Role Preferences of People with Multiple Sclerosis: Image-Revised, Computerized Self-Administered Version of the Control Preference Scale

**DOI:** 10.1371/journal.pone.0066127

**Published:** 2013-06-18

**Authors:** Alessandra Solari, Andrea Giordano, Jurgen Kasper, Jelena Drulovic, An van Nunen, Liina Vahter, Frederique Viala, Erika Pietrolongo, Maura Pugliatti, Carlo Antozzi, Davide Radice, Sascha Köpke, Christoph Heesen, on behalf of the AutoMS project

**Affiliations:** 1 Unit of Neuroepidemiology, Foundation Istituto Di Ricovero e Cura a Carattere Scientifico Neurological Institute C. Besta, Milan, Italy; 2 Department of Primary Medical Care, University Medical Center Hamburg-Eppendorf, Hamburg, Germany; 3 Institute of Neurology, School of Medicine, University of Belgrade, Belgrade, Serbia; 4 National MS-Centrum, Melsbroek, Belgium; 5 Department of Neurology, West-Tallinn Central Hospital, Tallinn, Estonia; 6 Department of Neurology, Purpan University Hospital, Toulouse, France; 7 Department of Neuroscience and Imaging, University “G. d’Annunzio” of Chieti-Pescara, Chieti, Italy; 8 Department of Clinical and Experimental Medicine, University of Sassari, Sassari, Italy; 9 Department of Neuromuscular Diseases, Foundation Istituto Di Ricovero e Cura a Carattere Scientifico Neurological Institute C. Besta, Milan, Italy; 10 Division of Epidemiology and Biostatistics, European Institute of Oncology, Milan, Italy; 11 Nursing Research Group, Institute of Social Medicine, University of Lübeck, Lübeck, Germany; 12 Institute for Neuroimmunology and Clinical MS Research (inims), University Medical Center Hamburg-Eppendorf, Hamburg, Germany; University Hospital La Paz, Spain

## Abstract

**Background:**

The Control Preference Scale (CPS) is the most frequently used measure of patients’ preferred roles in treatment decisions. We revised the original CPS and developed a new computerized patient self-administered version (eCPS). We used the eCPS to assess role preferences, and their determinants, in Italian and German people with multiple sclerosis (MS).

**Methods:**

New cartoons were produced, based on MS health professional and patient input/feedback and previous findings, and pilot tested on 26 Italian and German MS patients. eCPS acceptability and reliability (weighted kappa statistic, wK) in comparison to the original tool, was determined in 92 MS patients who received both CPS versions in random order.

**Results:**

The new cartoons were well accepted and easily interpreted by patients, who reported they based their choices mainly on the text and considered the images of secondary importance. eCPS reliability was moderate (wK 0.53, 95% confidence interval [CI] 0.40–0.65) and similar to the test-retest reliability of face-to-face administration assessed in a previous publication (wK 0.65, 95% CI 0.45–0.81). Higher education (odds ratio [OR] 3.74, 95% CI 1.00–14.05) and German nationality (OR 10.30, 95% CI 3.10–34.15) were associated with preference for an active role in the logistic model.

**Conclusions:**

The newly devised eCPS was well received and considered easy to use by MS patients. Reliability was in line with that of the original version. Role preference appears affected by cultural characteristics and (borderline statistical significance) education.

## Introduction

Over the last 15 years, initiatives to enhance citizens’ and patients’ influence in healthcare, and in particular to encourage shared decision making (SDM), have been proposed. These initiatives include empowering health providers to inform and involve patients; providing patient information and decision support systems [1]; and setting up patient education programs to prepare them for active involvement in decision-making [2]–[4]. Nevertheless, implementation of SDM in everyday practice is hindered by time and budget constraints, and also clinicians’ and patients’ attitudes, preferences, and expectations. Patient participation in medical care is generally considered to correlate with improved health outcomes [5]–[7]. However, studies indicate that while patients want more – and more accurate – information about their disease, their preferences regarding involvement in medical decisions vary considerably [8]–[11].

Therapeutic options for people with multiple sclerosis (PwMS) have expanded significantly in recent years. Long-term treatment with first-line ‘disease-modifying’ drugs is increasingly proposed soon after diagnosis. These treatments are only partially effective and associated with life-style changes and side effects, resulting in high dropout rates [12]. More effective and easier to administer drugs are also available, but these are more costly and associated with rare but severe side effects [13], [14]. For these reasons, decisions about starting or changing treatments for MS can be difficult for both patients and physicians, and interventions to increase patient involvement in decisions about their treatments and improve MS knowledge, confidence, and satisfaction with the decision making process have been recently established [13]. In this context, it is important to elucidate patient preferences regarding their involvement in decision making. Patient role preferences may influence the effect of interventions to increase patient involvement in decision making, while the concordance between preferred role and actual role can be an outcome measure of such interventions [15]. Formal assessment of preference is also important because health professionals have limited ability to discern or elicit the level of involvement preferred by their patients, in MS and other medical conditions [16], [17]. Finally, comparison of role preferences across health systems can reveal cultural differences and provide valuable information for initiatives to improve patient-clinician communication.

The Control Preference Scale (CPS) is the most frequently used instrument to assess patient preferences for involvement in decisions about their health [18], [19]. In 2006 we linguistically validated the Italian CPS, and assessed the preferences of Italian PwMS. We found that Italian PwMS generally preferred a collaborative role, while about a third preferred a passive role, and only about 6% prefer an active role [20]. These findings contrast markedly with those of a German study which found that 40% of German PwMS preferred an active role in decision making [21]. In the wake of this surprising finding we initiated the international project “Autonomy preferences, risk knowledge and decision-making performance in multiple sclerosis patients” (AutoMS; www.automsproject.org) to compare patient role preferences and investigate implementation of the SDM model in six European countries. It also seemed advisable to develop an electronic self-administered version of the CPS (eCPS) to standardize test presentation, eliminate the need for an interviewer and data entry, and thereby facilitate comparison of CPS performance across countries. Furthermore, indications from the Italian study [20] and discussion with the CPS author [18] suggested that the CPS cartoons would benefit from re-design.

The present paper reports the production of new CPS card cartoons; the migration of the face-to-face to the electronic version (eCPS); assessment of the equivalence of the eCPS to the original test; and prospective evaluation of determinants of role preferences in Italian and German PwMS.

## Methods

### Ethics Statement

All the study patients gave written consent to participate. The protocol was approved by the Ethics Committee of the following hospitals: Foundation IRCCS Neurological Institute C. Besta, Milan, Italy; University Medical Center Hamburg-Eppendorf, Hamburg, Germany; University of Belgrade, Belgrade, Serbia; National MS-Centrum, Melsbroek, Belgium; Department of Neurology, West-Tallinn Central Hospital, Tallinn, Estonia; Department of Neurology, Purpan University Hospital, Toulouse, France; University “G. d’Annunzio” of Chieti-Pescara, Chieti, Italy; University of Sassari, Sassari, Italy.

### CPS Administration and Scoring

**Figure 1:**
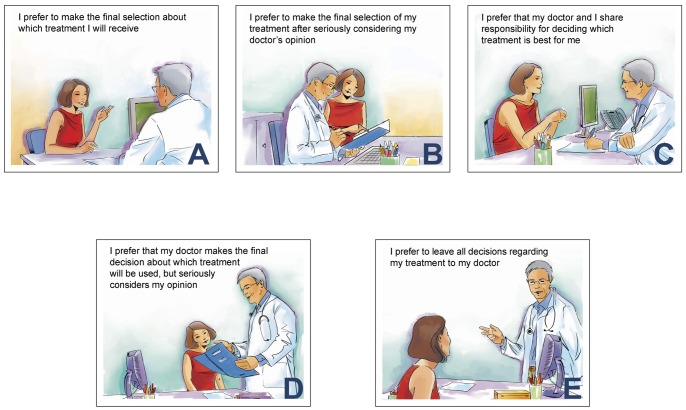
The five CPS cards with new cartoons.

The CPS was developed to evaluate the amount of control individuals want to assume in decisions regarding their health [18]. It consists of five “cards” on a board, each illustrating a different role in decision-making by means of a cartoon and short descriptive statement (Figure 1). The examiner asks the respondent to choose the preferred card, which is then covered up and cannot be chosen again; the examiner then asks the respondent to choose the preferred card from the remaining four cards. The procedure continues (four choices) until one card is left. If the second preference is incongruent with the first (non adjacent pairing, such as card A with card C), the test is explained again, and immediately re-administered. In the event of a further incongruence, the test is not re-administered, and a preference is not assigned. Administration requires about 5 min. Six scores are possible based on the subject's two most preferred roles: active–active, active–collaborative, collaborative–active, collaborative–passive, passive–collaborative, and passive–passive. These scores are grouped as: active (active–active or active–collaborative), collaborative (collaborative–active or collaborative–passive), or passive (passive–collaborative or passive–passive).

### Production and Evaluation (Cognitive Debriefing) of New CPS Cartoons

A professional cartoonist working in medical publishing was selected from three applicants. A panel consisting of the cartoonist and the three persons (neurologist, psychologist and lay person) who had validated the Italian CPS were involved in producing the new cartoons. At the first panel meeting, the original instrument and comments of PwMS who took part in the previous study [20] were presented to the cartoonist and discussed. A month later the cartoonist submitted her first set of cartoons to a reconvened meeting. Following discussion, the cartoonist revised the cartoons. Revisions were presented and re-revised at further meetings until no further changes were suggested. Finally, the cartoonist further revised the cartoons taking into account feedback from AutoMS investigators.

The acceptability and clarity of the “new” CPS (new cartoons plus original captions) was assessed in a minimum of 10 cognitive debriefing interviews with Italian and German PwMS aged ≥18, with relapsing-remitting course. Patients with exacerbations in the previous month, definite cognitive compromise, any compromise precluding participation (e.g. severe visual impairment), or those who had already received the CPS were ineligible. Each patient was administered the new CPS. There followed a series of open-ended questions on the clarity and utility of the instrument as a whole and each new cartoon, based on an interview guide previously drawn up and agreed by the German and Italian investigators. By means of a 0–10 visual analogue scale (VAS), patients were asked to assess the extent to which the CPS expressed their attitudes to involvement in decisions (a) on therapy in general, and (b) on MS immunotherapy.

### eCPS Production, Reliability and Usability

The eCPS was designed such that, on a monitor of 15 inches or more, the electronic cards were similar, in terms of size and color, to the new CPS cards. The mode of presentation of the electronic cards and performance of the test matched the original [20]. The eCPS test was preceded by a multiple choice socio-demographic information screen to be completed by the patient; basic clinical information was subsequently added by the clinician.

The reliability of the eCPS compared to the face-to-face version was assessed on at least 80 PwMS (Italians and Germans) with relapsing-remitting MS aged ≥18 years, excluding those with exacerbations in the previous month, definite cognitive impairment, and any compromise precluding participation (e.g. severe visual impairment). One of the tests was given to each patient; the other test was administered 4–6 weeks later (crossover design, random test order). This interval was considered long enough to obviate recall, and short enough so that a change in the patient’s condition (or role preference) was unlikely (any clinical exacerbations between the two tests were recorded). A sub-sample of 20 patients assigned to receive the eCPS after the original CPS, were cognitively debriefed immediately after eCPS administration to obtain feedback on eCPS acceptability and usability, using a previously drawn up interview guide.

### Statistical Analysis

Variables were summarized by means with standard deviation (SD), or medians with interquartile ranges (IQRs) or ranges. Categorical variables were compared using the chi-squared test, and continuous variables using Kruskal–Wallis ANOVA, or Wilcoxon test for two independent samples. eCPS reliability (crossover design) was assessed on the six preference categories with the weighted kappa statistic (wK) with 95% confidence intervals (CI) estimated by the bootstrap method (5000 replicates). We used the following weightings: 1−|i−j|/(k−1), where i and j index the rows and columns of the ratings at the two administrations, and k is the maximum number of possible ratings [22]. We estimated that a minimum of 80 subjects was required to obtain a kappa value of at least 0.50 (null hypothesis: kappa = 0.30) under the following assumptions: alpha = 0.05 (two-sided); power = 0.80; two ratings (tests); six categories with uniform frequency distribution.

Logistic regression was used to assess the influence of the pre-specified explanatory variables (age, sex, education, length of follow-up at participating center, and country) on eCPS active role preference (vs. collaborative or passive). Continuous variables were dichotomized with medians as cut-offs. Model goodness-of-fit was investigated by the Hosmer–Lomeshow test [23]. Results are reported as odds ratios (OR) with 95% CI. Data were analyzed using Stata Statistical Software, release 12 (Stata, College Station, Texas). All tests were two-tailed and p values <0.05 were considered significant.

## Results

### Production and Evaluation (Cognitive Debriefing) of New CPS Cartoons

The new cartoons were produced between May and September 2010. Unlike the originals they were colored and were also considered to be more modern (Figure 1). The thought bubbles in four of the original CPS cards, and the hand-shaking on card C were no longer present: this rendered the cards more uniform. Because the new images were more detailed, new problems arose regarding the gender and age of the patient and the physician, and the nature of the background. The panel agreed on a female patient and male physician, and a minimal background compatible with a hospital/outpatient consultation room. A version with images portraying a male patient was also produced intended for use in male health conditions (Figure S1).

After discussion among AutoMS investigators, the following changes were made: (a) The patient’s expression was changed on Card A, so that she now appeared less frivolous and to be actively speaking. (b) Contact between the physician’s hand and patient’s shoulder was removed from Card E. (c) On card E, the focus was shifted to the physician instead of the patient, whose back was now turned, so that this card mirrored card A (where the focus is on the patient with the physician’s back turned).

Between December 2010 and January 2011, 26 MS patients received the “new” CPS: 16 were from three Italian tertiary MS referral centers, and 10 from the Hamburg outpatient MS clinic. Twelve (46%) were women; median age was 37 years; median disease duration was 9.3 years; and median EDSS score 2.5 (Table 1). Three Italian MS patients gave incongruent answers on both the first and second administration, so CPS scores were not obtained. CPS scores were therefore available for 23 (88%) participants: 10 (44%) preferred a collaborative role; 9 (39%) an active role; and 4 (17%) a passive role. Role preference differed significantly between countries, with active/collaborative/passive roles reported by 15%/55%/30% of Italian vs. 70%/30%/0% of German patients (p = 0.016).

**Table 1:** Characteristics of the patients participating in the two study phases.

Characteristic	Sub-characteristic	Phase I: new cartoons		Phase II: eCPS reliability	
		Germany (N = 10)	Italy (N = 16)	Germany (N = 38)	Italy (N = 54)
Women (%)		3 (30)	8 (50)	26 (68)	33 (61)
Age (years)^1^		42, 12 (26–63)	36, 7 (27–48)	39, 10 (18–62)	38, 9 (19–55)
Time from first symptoms (%)	≤5 years	0 (0)	0 (0)	13 (34)	19 (35)
	>5 years	10 (100)	16 (100)	25 (66)	35 (65)
Setting (%)	Outpatient clinic	0	9 (60)	26 (68)	25 (46)
	Day-Hospital	10 (100)	6 (40)	12 (32)	29 (54)
Education (%)	Primary (5–10 y)	2 (20)	3 (19)	15 (39)	10 (19)
	Secondary (11–13 y)	4 (40)	4 (25)	9 (24)	29 (53)
	College/University (≥14 y)	4 (40)	9 (56)	14 (37)	15 (28)
Work (%)	Employed full-time	5 (50)	10 (62)	12 (31)	31 (57)
	Employed part-time	1 (10)	0	5 (13)	4 (7)
	Unemployed	0	2 (12)	5 (13)	5 (9)
	Student	1 (10)	2 (12)	3 (8)	3 (6)
	Homemaker	1 (10)	1 (6)	3 (8)	5 (9)
	Retired	0	0	1 (3)	0
	Disability-support pension	2 (20)	1 (6)	9 (24)	6 (12)
EDSS score^2^		3.0 (0.0–6.0)	2.2 (0.5–6.0)	3.0 (2.5–4.0)	2.0 (0.0–6.5)
Disease-modifying treatment (%)	Interferons	0	6 (37)	2 (7)	4 (9)
	Glatiramer acetate	0	4 (25)	1 (4)	14 (31)
	Natalizumab	3 (30)	5 (31)	25 (89)	20 (43)
	Immunosuppressants	2 (20)	0	0	8 (17)
Followed at MS center (%)	<1 year	5 (50)	3 (19)	9 (24)	5 (10)
	1–5 years	4 (40)	3 (19)	19 (50)	19 (35)
	>5 years	1 (10)	10 (62)	10 (26)	30 (55)
Use of computer^3^	No	NA	NA	0	5 (10)
	Rare (less than once a week)			2 (6)	4 (8)
	Moderate (about weekly)			2 (6)	2 (4)
	Frequent (more than weekly)			32 (89)	39 (78)
Test-retest interval (days)^2^		NA	NA	28 (14–56)	34 (23–59)

CPS is Control preference Scale; EDSS is Expanded Disability Status Scale; NA is not applicable.

1 Mean, SD (range).

2 Median (range).

3 Missing information: n = 2 Germany; n = 4 Italy.

In general, the cartoons were well received and considered clear, but almost all patients considered they had a minor role compared to the text, indicating that their choice was mainly based on the text: “You could have omitted the cartoons. They are nice but not enough on their own to allocate role preference” [German patient]; “They [the images] are a nice accompaniment, they don’t distract; they remind me of children’s books” [Italian patient]; “I didn’t mind the pictures; they didn’t distract me from the text… they are appealing and appropriate” [German patient]; “I just used the text. The pictures were secondary, not to be taken seriously, I could have read a comic instead… They are interchangeable” [German patient].

All patients reported that it was easy for them to pick one of the five cards that best described their preference. One Italian patient commented that, by choosing more than one card, the patient strengthens the first choice with the second.

Patients assigned a median VAS score of 8.0 (IQR 7.5–9.0) for the extent to which the CPS expressed their attitude to involvement in decisions about therapy in general. The VAS score for involvement in decisions on immunotherapy was 9.0 (IQR 8.0–9.0). Regarding the issue of acceptability, considering that the patient in the cartoons was a woman, a male patient found this acceptable as the “woman with MS has to do with disease statistics”; the other patients reported that they did not notice or were not bothered.

### eCPS Production, Reliability and Usability Testing

Between October 2010 and May 2011, the electronic version was produced in English, German and Italian, and the screens for recording responders’ socio-demographic and basic clinical information inserted.

Between August 2011 and March 2012, 92 MS patients, 54 in Italy, and 38 in Germany (Table 1) participated in the crossover study. Four participants (three Italian) gave incongruent answers, and one Italian patient did not perform the second test: Full CPS scores were therefore available for 87 (95%).

Median test-retest interval was of 4 weeks in Germany and 5 weeks in Italy. In Germany there were three protocol violations (patients on steroids for an exacerbation at enrolment); these were included in the analysis. Of the included patients, 71 (83%) used a computer more than once a week, and 63 (72%) had internet at home.

**Figure 2:**
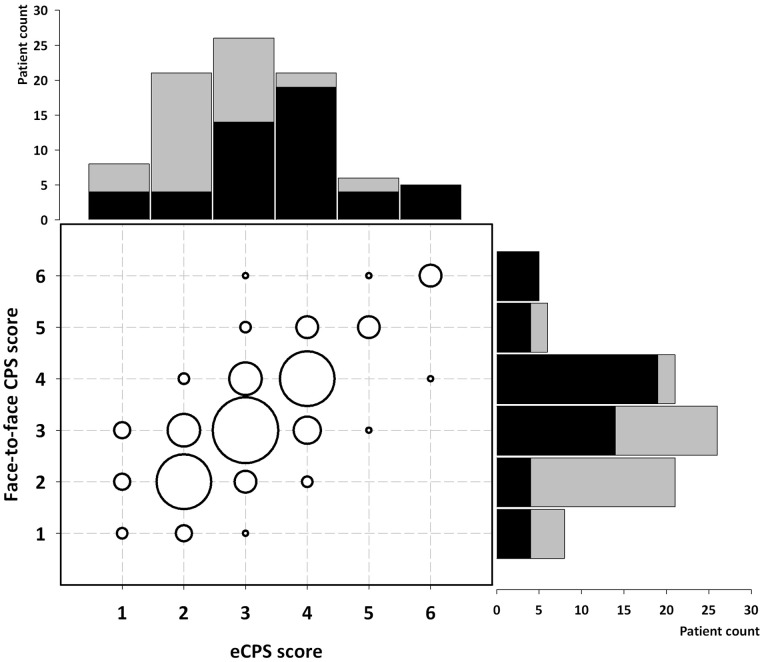
Distribution of role preferences according to administered CPS (face-to-face vs. electronic, eCPS) in 87 MS patients. CPS scores range from 1 (active-active) through 6 (passive-passive). The histograms report numbers of patients with each CPS score, by country (Italy, black; Germany, grey). The bubble plot shows pairs of counts of each score, with larger bubbles corresponding to higher counts.

Role preferences by administration (face-to-face vs. electronic) and country are shown in Figure 2. The reliability of the eCPS in relation to the face-to-face CPS was moderate: observed agreement was 85%, wK 0.53 (95% CI 0.40–0.65; p<0.001). These findings are consistent with the test-retest reliability of the face-to-face CPS obtained in Italy (observed agreement 90%; wK 0.65 [95% CI 0.45–0.81]; p<0.001) [20]. Five participants did the eCPS test twice because of inconsistent choices, two of the five made inconsistent choices at second administration (invalid test). Two participants did the face-to-face CPS twice for the same reason, and both made inconsistent choices at second administration.

From interviews with 43 patients who received the eCPS after the face-to-face version, the tool was found to be well accepted by 41 (95%) and considered useful by 39 (90%). Twenty-two patients (51%) said they based their choice on the text only, 14 (32%) mainly on the text, and 7 (16%) on both text and images. With regard to eCPS usability, 39 (90%) considered the instructions easy to understand and the overall procedure user-friendly: one 33 year-old man with tremor had difficulty using the mouse; two women (of 55 and 37 years, both with elementary education and unfamiliar with a computer) had some difficulties navigating.

Patients assigned a median VAS of 8.0 (IQR 7.0–9.0) to the extent to which the CPS expressed their attitudes to involvement in decisions both about therapy in general and about immunotherapy.

### Role Preference Across Countries

We used logistic regression to assess factors associated with active role preference (Table 2). German PwMS were seven times more likely to prefer an active role than Italians (OR 6.89, 95% CI 2.54–18.68). This association was strengthened (OR 10.30, 95% CI 3.10–34.15) after controlling for gender, age, education and length of follow-up at the MS center. Higher education was positively associated with active role preference (OR 3.75, 95% CI 1.00–14.05). Length of follow-up at the MS center for five years or more was negatively associated with active role preference in the univariate analysis (OR 0.35, 95% CI 0.13–0.93), but this association was no longer significant after controlling for the other explanatory variables (Table 2). Neither gender nor age were associated with active role preference.

**Table 2:** Variables associated with active role preference on the eCPS among 87 people with MS in univariate and multivariate models. Age was categorized into two classes, with median as cutoff.

Characteristic	Sub-characteristic	No at risk, Event (%)	Crude OR (95% CI)	*p* value	Adjusted OR (95% CI)*	*p* value
Age (years)	<38	44, 16 (36.4)	1	0.54	1	0.87
	≥38	43, 13 (30.2)	0.76 (0.31–1.86)		0.91 (0.31–2.72)	
Sex	Men	30, 11 (36.7)	1	0.63	1	0.35
	Women	57, 18 (31.6)	0.80 (0.31–2.02)		0.59 (0.20–1.78)	
Education	Primary	22, 6 (27.3)	1	0.48	1	0.05
	Secondary or higher	65, 23 (35.4)	1.46 (0.50–4.24)		3.75 (1.00–14.05)	
Followed at the MS center	≤5 years	49, 21 (42.9)	1	0.03	1	0.39
	>5 years	38, 8 (21.1)	0.35 (0.13–0.93)		0.61 (0.19–1.88)	
Country	Italy	50, 8 (16.0)	1	<0.001	1	<0.001
	Germany	37, 21 (56.8)	6.89 (2.54–18.68)		10.30 (3.10–34.15)	

OR is odds ratio, and 95% CI the OR confidence interval, estimated by unconditional logistic-regression.

Multivariate model including all explanatory variables, with Hosmer-Lemshow goodness-of-fit test χ^2^ = 7.7 (degrees of freedom = 22), *p* = 0.47.

## Discussion

When a qualitative approach is not feasible, the CPS is useful to assess the role preferences of patients and citizens in large-scale studies [24]. Administering the CPS on a computer improves the standardization of test presentation and reduces investigator involvement. However, eCPS equivalence with the original version and receiver acceptability require careful assessment. Recently, both computerized and video CPS versions have been used in patients with prostate cancer [25]–[27]. The video CPS proved feasible and acceptable [28], but the equivalence of these new versions with the original test was not investigated.

We found that both Italian and German MS patients received the eCPS well and found the instrument easy to understand. In our crossover sub-study, concordance of the eCPS with the face-to-face version was moderate, and in the same range as the test-retest reliability of the original version [20], indicating that concordance was unaffected by mode of administration, and supporting the equivalence of the eCPS with the original test [29]. Test usability was very good, with all participants completing both the questionnaire on the opening screen and the eCPS test, about a quarter of whom only had elementary education, while 12% used the computer rarely or not at all. Both Italian and German participants based their choices mainly on the text and considered the images of secondary importance. Opinions differed, however, about the utility of the images: Italians were more positive about them, suggesting they complemented the text, while Germans were less enthusiastic, some regarding them as superfluous or irrelevant.

In line with our previous findings, we found a marked difference between Italian and German participants with respect to decision-making preference, in that German MS patients wanted a more active role [20], [21]. Importantly, this finding was independent of gender, age, education and length of follow-up at the MS center. In agreement with studies in other health contexts, well educated participants were also more likely to prefer an active role in the multivariate model (borderline significance) [8], [9], [30], suggesting that education facilitates patient empowerment. In contrast to findings that women and younger people generally prefer a more active role [8], [10], neither gender nor age had an influence on role preference in our study.

The fact that Italian PwMS prefer a collaborative or passive decisional role is not at odds with their wish to be more informed about their disease from the moment of diagnosis communication [31]. Other studies also indicate that while patients want more – and more accurate – information about their disease, their preferences regarding involvement in medical decisions vary considerably [8], [30].

Data from all six countries participating in AutoMS are currently being collected: preliminary findings that include data from Serbia and Estonia confirm that German PwMS significantly prefer an active role, while Serbian and Estonian participants do not differ significantly from each other or Italy (reference) (data not shown). Country-specific variations in health system organization might contribute to these differences; in particular it is known that recommendations on patient and citizen empowerment started being implemented earlier in Germany than other European countries [2]–[4]. However, discerning the influences of health system characteristics and SDM promotion initiatives is not straightforward [5]. Moreover, social and policy changes can take time to affect everyday practice, as shown by a recent study showing that, more than 20 years after unification, people in former Eastern Germany prefer more passive roles than those living in Western Germany [32]. A recently published study in Hispanic patients with advanced cancer found that preference for a passive role was four-fold higher in those living in Latin America compared to those living in the US, after controlling for age and education [33].

Our study was confined to patients with MS, most of whom were young females (typical of the condition): our findings are therefore unlikely to be applicable to other illnesses or populations. In addition, while the Italian participants were from three geographically disparate areas (Northern, Central and Southern Italy) the German participants were all from the area of Hamburg which may not be representative of Germany as a whole.

Another limitation is that we did not take account of other characteristics that may influence variation in patient decision-making preferences, such as socio-economic status, depressive symptoms, proximity to the decision about whether to start or change immunotherapy, and theory-based behavioural measures [34], [35]. In this regard, we have recently devised a patient questionnaire on MS immunotherapy decision making, based on the theory of planned behaviour [36]. This questionnaire has been translated and culturally adapted into the AutoMS languages (paper in preparation) and will be used as moderator of role preferences (assessed with the eCPS) in an international online survey of PwMS considering whether to start or change immunotherapy.

## Supporting Information

Figure S1 **New CPS cards for use in male health conditions.**
(TIF)
