# Supplementary figures and images for: Role Preferences of People with Multiple Sclerosis: Image-Revised, Computerized Self-Administered Version of the Control Preference Scale
Source: PLoS One. 2013 Jun 18;8(6):e66127. doi: 10.1371/journal.pone.0066127 (PMC3688863; doi:10.1371/journal.pone.0066127)

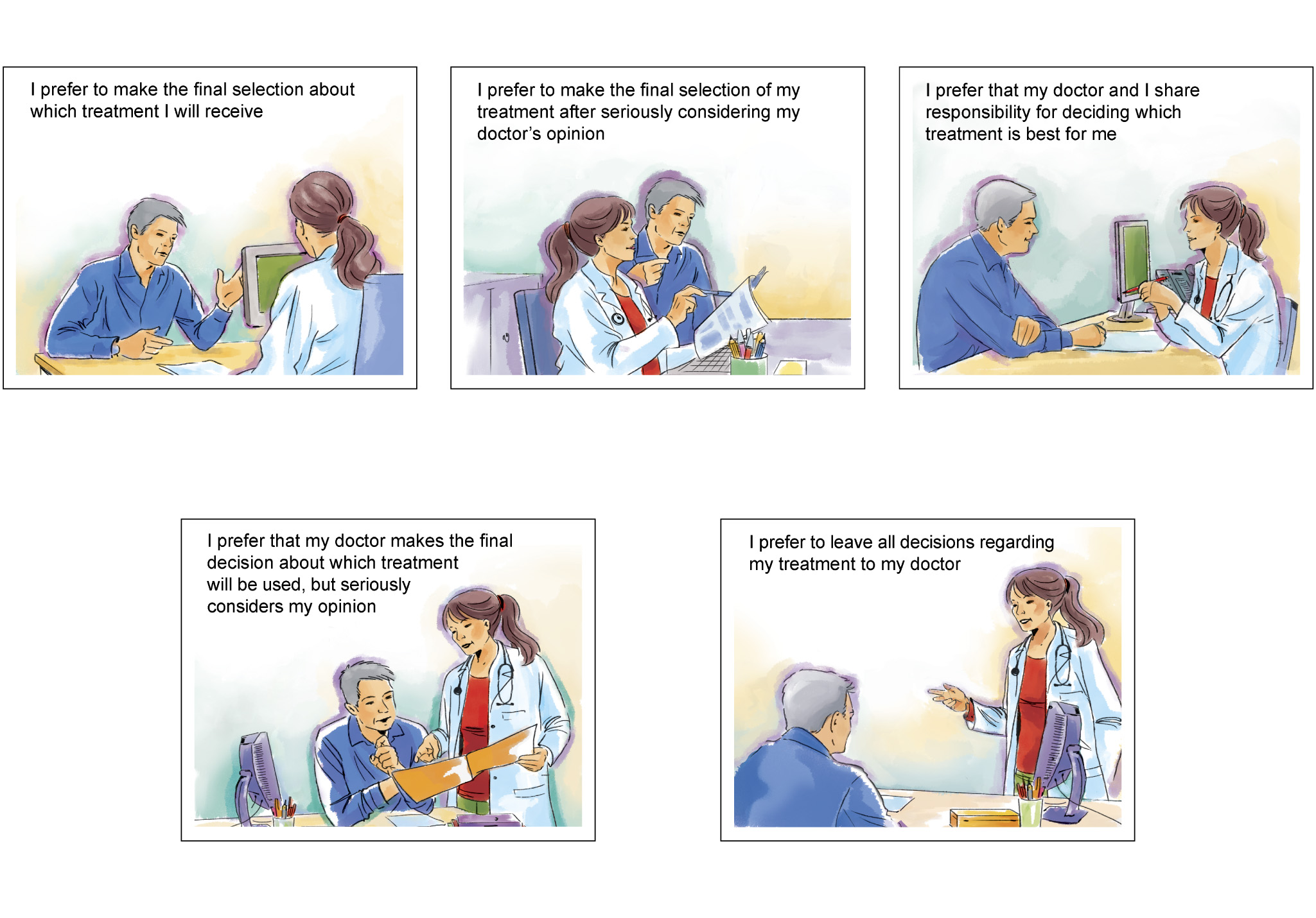

Supplement: Figure S1 — New CPS cards for use in male health conditions. (TIF) [file pone.0066127.s001.tif]
